# UIOT-FMT: A Universal Format for Collection and Aggregation of Data from Smart Devices

**DOI:** 10.3390/s20226662

**Published:** 2020-11-20

**Authors:** Mats Seljeseth, Muhammad Mudassar Yamin, Basel Katt

**Affiliations:** Department of Information Security and Communication Technology, Norwegian University of Science and Technology, Teknologivegen 22, 2815 Gjøvik, Norway; matsse@stud.ntnu.no (M.S.); basel.katt@ntnu.no (B.K.)

**Keywords:** IoT, smart city, law enforcement

## Abstract

Information Technology (IT) has become an essential part of our lives and due to the emergence of the Internet-of-Things (IoT), technology has encompassed a majority of things that humans rely on in their daily lives. Furthermore, as IT becomes more relevant in daily lives, the need for IT to serve public emergency services has become more important. However, due to the infancy status of IoT, there is a need for a data consortium that would prove to be best used in servicing policing in a technological driven society. This paper will discuss the plausibility of creating a universal format for use in carrying out public services, such as emergency response by the police and regular law maintenance. In this research we will discuss what the police requires in their line-of-duty and how smart devices can be used to satisfy those needs. A data formatting framework is developed and demonstrated, with the goal of showing what can be done to unifying data from smart city sensors.

## 1. Introduction

The realm of Information Technology has spurred a renaissance in our society, where our day-to-day devices are becoming interconnected and thus becoming smarter in a way that they communicate and perform their designated tasks. Manufacturers of commercial appliances strive towards giving its consumers the best products conceivable, thus promoting more to enable their products the capabilities of communicating over the Internet. This paradigm shift has impacted almost all aspects of our society, leaving only a few communities unaffected by this paradigm. One particular area of our society that has yet to see a shift towards smarter use of appliances is the law enforcement profession. There has only in recent years been plans to develop appliances for use in the police profession. Proposals for the use of autonomous vehicles and robots to aid law enforcement operators in the field were only introduced in recent years. However, there is an untouched area of smart technology that has yet to be used in law enforcement. Data that is constantly being generate by smart devices can potentially provide field operators and forensics staff with information that would tell more about a criminal incident/case. In [1] researchers proposed that any form of data captured on an IoT sensor could potentially be used for collection and analysis. Such capabilities of this data makes it theoretically possible to use IoT sensor data in a *Computer Network Intrusion Detection* [2]-like manner. It is unfortunate, that the feasibility of conceiving such a system is inhibited due to the diversity of devices that are available on the market today. Manufacturers and developers are at a disagreement of what data and communication standards should be implemented in their devices.

According to [3] this has led to an IoT landscape, in which the devices are unique from one and other in a way that they are being “Smart”. Communication and data format are therefore the main obstacle from implementing a mass monitoring system in a smart city. The goal of this research is to develop a data formatting framework for the use in law enforcement. The focus of this format is to provide its users with the capabilities of selecting what information gets collected and how it is handled after being collected. With such a novel concept being developed using a modern technological landscape to enhance the capabilities of law enforcement, there are a few issues that must be addressed in order to progress in this area. This research will focus on three main problems relating to how a city wide policing system is to be developed. As a starting point for creating the format for a smart policing platform the following concerns must be addressed.

1. What type of information would be necessary for law enforcement agencies to carry out their tasks in a proactive manner?
2. What category of IoT devices in a Smart City can be leveraged by law enforcement to obtain useful knowledge of on-going crimes?
3. What data format structure of high-level data will ensure that data output can be processed from different devices and outputted in a unified format?

The main contribution of the research work is the development of an artifact for data unification and how it is implemented to handle the challenges of data heterogeneity. An evaluation of the proposed solution is conducted by comparing the results of this work against competing standards. For the evaluation, the main focus will be to measure the reliability and performance of the artifacts and compare them against its competitors. Rest of the paper is structured as fallows, first, we provide a brief research methodology and related work. Continuing that we will present police needs and diversity of IOT devices gathered from literature surveys and interviews. After that we will present the proposed system, its evaluation and finally we will conclude the article with conclusion and future work.

## 2. Research Methodology

Design science Research that was introduced in [4] is the chosen methodology for this thesis. The main goal of this methodology is to develop artefacts to solve real world problems and add new knowledge, through a process of (1) understanding and identifying the problem, (2) designing the solution, (3) developing the artefact, (4) evaluating the developed proposed artefact, and (5) communicating the resulted knowledge. In the beginning of this process the current knowledge associated with the problem domain is used to understand and identify the problem to be solved, and at the end, insight to the problem domain and new knowledge is generated and added in a feedback loop. This process can be done in iterations in order to improve the developed artefact. The reason DSR is an ideal methodology for this research, is that the development of software will always require further improvements to be made. Software artefacts are never fully developed on the first iteration, and there are always ways for improvements. This methodology is therefore ideal to address how software could be a solution, and how its shortcomings can be improved upon.

## 3. Related Work

In the area of research on the unification of smart device and smart sensor data, many research papers were written. Given that the Internet of Things is a recent phenomenon, the degree to which research has further progressed this phenomenon is extensive. However, given that the age of IoT is still recent, the amount to which the standardization of some aspects remains untouched. This can be seen in the vast number of devices that exist today and manufactured by small and large companies, who all sought to gain a market value. In achieving so, a lot of proposed standards have been published alongside the devices that merged during the rise in popularity of IoT devices. While one device might use a wireless protocol such a Zigbee, another similar device might use a protocol such as Z-Wave or Bluetooth Low-energy (BLE). Another issue that arose during the IoT-boom was the disagreement revolving around what the devices were to communicate between each other, which led to the creation of a diverse pool of data formats. Formats that spans along various byte-orders and serializations, which makes the idea of interoperability difficult. In order to develop a system in which a police force is to collect data from smart devices, a standardization must be established.

For the sake of developing a data unification framework in pursuit of crime monitoring, the amount of research is sparse. Mainly, the research activities that revolve around the development of a unified framework is more focused on the developing such a framework for commercial and industrial use. For instance, in [5], a novel data aggregation model was proposed for use on environmental sensors in a smart city. The focus of their research encompassed the use of network sensor data from industrial-based sensors, which monitors water, electricity and gas-based sensors. A more concrete implementation of a model that can be used to translate smart city sensor data was proposed in [6], where they demonstrated how the North Atlantic Treaty Organization (NATO) can leverage smart city appliances in urban operations. In disaster situations that takes place within an urban environment, the research group believe that the integration between IoT devices in a smart city environment could be integrated with the systems in a Federated Mission Network (FMN). This integration was proposed to aid soldiers in obtaining intelligence for use in their vehicle systems, urban personnel deployment and *Unmanned Aerial Vehicle UAV* systems. What makes this paper relevant for this project is that it outlines a few similar ideas that are aligned with the notion promoted in this research: to use peripheral data sources in pursuit of a safer execution of tasks in an urban environment.

In [7], a novel way of integrating crime incidents and police vehicle locations in a smart city was proposed. This system used smart technology in conjunction with police vehicles in order to supply its officers with information about the crime in advance. It is not strictly a research into the use of smart city data, but the paper does illustrate a good point on how technology is used to effectively dispatch units. Moreover, the project also discussed a relevant point in regard to the use of *Global Information System GIS* technology to improve the logistics of dispatching units. Whereas the city is divided into areas, where the crime model bases itself on the location where crime is the most frequent. This type of approach to distributing crime events based on the location where they occurred, could be an ideal way of establishing logistics for a smart policing system.

In [8], researchers explored the prospect of using data mining techniques on heterogeneous data to provide law enforcement with a bigger picture of the incidents, that takes place within the city of Newark (New Jersey). They focused more on integrated cooperation between precincts and the use of government registries to improve crime fighting, but their ideas still shares the same sentiments towards the use of data mining to aid in the process of ensuring public safety. Further elaboration on the potential for data fusion of smart city data is discussed in [9], where a comprehensive survey is conducted on the topic of IoT and Data Fusion. The paper provided an adequate overview over for the requirements of fusion of data in a Smart city environment. Furthermore, the researchers also shared data from common devices found in a smart grid, supplied with information pertaining to the category of the data.

In [10], researchers covered several important concepts in correlation to the Internet of Things and assembling data into a new set that can provide the consumers with more information. In conjunction with data fusion concepts, the researchers focused on ideas, such as the construction of incidents, based on the readings gathered from smart city devices. Moreover, they also focus on context awareness and its importance in IoT to supply more information about events, using multiple data sources. As a result of this survey, they propose an evaluation framework for data fusion with 10 points covering their core topics. Another unified format for smart sensors was proposed in [11], where the authors translated data from protocols such as CoAP, 6LowPAN, UDP, 802.15.4 to detect anomalies in smart city sensors. Location tracking technologies were used in conjunction with data from these sensors, to detect anomalous readings in humidity, light, Carbon Dioxide (CO2) levels. Furthermore, the data were placed in a timeline using timestamps from the data entries, such that these data metrics were properly mapped by time.

In [12], researchers proposed a Stream Annotation Ontology model (SAO). This data format was introduced as a part of the smart city project CityPulse and illustrated a different way of structuring time-series data from open data repositories that is supplied by the city of Aarhus in Denmark. As noted in the paper, the main focus of city pulse is the use of open traffic events with timestamps and location data to form a bigger picture of incidents that takes place in Aarhus. A formatting standard for use in sharing evidence artefacts that originates from IoT devices were introduced in [13]. Their data format was presented as a means to which law enforcement could share their findings and experiences from working with IoT devices. Law enforcement lacks the ability to efficiently share evidence and experiences from working with IoT devices. Therefore, [13] developed this format for the sole purpose of allowing *Law Enforcement Agencies* LEAs to share this information in a manner that does not reveal sensitive information and also allows for an easier way of reading the accumulated sensor data.

## 4. Police Needs

Law enforcement can be divided into two groups: first is the group that aims to deter, alleviate and prevent crime in society and there is the group who is responsible for ensuring that the law is practiced in a just manner. These two groups makes up the core responsibilities of a Law Enforcement Agency in Norway. The first of which is referred to a patrols or field-operators, as this group consist of the men and women that are actively in the field, fighting and preventing crime as they occur. The second group will be referred to as forensics, since their responsibilities are to procure and analyze data as they are discovered at the scene of the crime.

### 4.1. Field-Operator’s Needs

A field operator is the active force of men and women, who are out in the cities, suburbs and rural areas to protect citizens from harm and loss of property. Their daily routines consist of unpredictable encounters and situations, that put these operator’s lives in danger to ensure that other’s lives are protected.

#### 4.1.1. Intelligence

The use of intelligence is arguably not a new practice in risk-related professions. In activities, such as wildlife preservation, military operations, geological excavations and so on, there is an element of prior knowledge, obtained by its participants, in light of executing their respective tasks. Obviously, the tasks being carried out in this manner, is done so, to reduce a reaction from occurring as a result of the work. It is considered in some way, a risk assessment of the underlying and/or peripheral environment, in order to arrive at the conclusion, on whether the ensuing task/challenge will cause an unwanted event to occur. Military work often uses the term intelligence as a term for information obtained on relevant adversaries that could aid in gaining a significant advantage over said contenders [14]. In geology, the use of intelligence could be in the form of a conducted risk assessment of a geological site, to assess the likelihood of there being hazards being present [15]. With no regard to the nature of the profession, whoever uses any form of intelligence, the general census for using such methods, is to improve upon the quality of the job-outcome, or purportedly to reduce the likelihood of accidents and injuries. With respect to the police profession, the use of intelligence is postulated to be a deciding factor, which could affect the outcome of an officer’s work day, as well as their prowess. Use of intelligence is doubtfully a novel concept for law enforcement either; given that use of information to point patrols in the right direction has been practiced for decades with the emergence of telecommunication, such as seen in the use of emergency hotlines.

#### 4.1.2. Deterrence

The previous section have discussed the possibilities of lifting the quality of police work by reducing risks and improving information flow, there are other potentials ways which can be exploited in a smart city environment. This part does not include the quality of the information that could improve the police officer’s work efficiency and safety, but rather reduce the number of crimes to which they are required to respond to. In order to reduce the number for crimes within a metropolitan environment, there must be an element present that discourage or deter the perpetrator. In a smart city there could be a potential for employing various types of devices, which possesses the capability of warding off potential criminals from carrying out their heinous acts, by simply being installed and present. In other words, some smart devices, when configured appropriately, can serve the public by acting as a means of which the crime does not take place, due to the possibility of the crime being recorded by the device’s sensors. There exist at this time various implementations of IoT devices and *Information and Communication Technology* ICT devices which can have this effect on the general public, by simply being present and visible to the public.

### 4.2. Digital Forensics

Digital forensics is another aspect of law enforcement which could benefit from using data from smart devices to investigate a crime. While digital forensics are a well established field of law enforcement, there are other unexploited data sources which could be used to further infer the extent to which a suspect is guilty. What sets forensics apart from patrol-duty is the fact that the work undertaken by forensics staff is oriented towards answering the questions left behind, prior to the completion of the crime. Police patrols main tasks is the prevent and intervene in an ongoing crime and does so through direct intervention, rehabilitation (Extent to which rehabilitation of criminals, depends on a country’s legal system) and deterrence. A crucial distinction must be made when addressing the need for law enforcement, as these two working-groups require disparate requirements to do their job. Forensics are an important part of law enforcement which enables the juridical system to fill in the blank areas, that is the untold stories of a crime, which could play a crucial role in providing the evidence that would enable the law to be served to the best of its ability. Forensics are a significant element in juridical process, where forensic analysts conduct investigation of a crime scene. Analysts in forensics process would observe the scene of the crime for the evidence which could fill in the blanks of a criminal case, such that the appropriate legal action can be taken against the perpetrator. All work processes in a forensic investigation differs from how the field related duties are laid out, meaning that the main focus is not on being able to apprehend the suspect safely, nor is it to be able to prevent a crime. Rather the main focus of a forensic analyst is to be able to provide evidence that could aid in an investigation, such that the suspect(s) are apprehended and punished. In order to achieve this goal a forensic investigator has to examine the evidence that relates to the case and in order to do so they have to acquire such evidence.

### 4.3. Identify the Source of the Digital Evidence

In IoT the *intermediate layer* represents the devices that acts as a connection between smart sensors and the cloud. For devices and sensors who communicates wireless, through communication protocols such as 6LowPAN, Bluetooth, Z-wave, Zigbee, 802.15.4, LTE or LoRA, have to connect to an intermediary element before the data can reach the cloud. For the devices who relies on wired connection the case is the same, as they would also require some sort of connecting relay. Ref. [16] Some devices that are a connecting medium includes, conventional computers, smartphones, proprietary smart hubs, routers and gateways. When it comes to intermediate level, the devices including phones, computers can be approached using conventional computer forensics. This is due to the fact that research into investigating these instances of hardware is well documented and commonly practiced. When obtaining access to these devices, the only requirement is that the credentials to the controller is available. However, with the inclusion of mediums, such as gateways, smart hubs and routers, the availability of the evidence relies on whether or not the manufacturer allows its users to access their hardware. In [17] some devices that communicates with a hub over wireless protocols can be intercepted, using special hardware that communicates with the same protocol as the device in question. However, depending on the device, this data could be encrypted. In some cases, the data is not encrypted at all. How the data is assembled on the device before being sent to the hub is another problem with device to intermediary interception. If the data is assembled in a way that is not understandable, then additional steps are required to reverse engineer and implement a decoder for every device who sends data this way. According to Samsung’s SmartThings documentation [18] on Zigbee, illustrates how a zigbee Join request is assembled. At first glance, their implementation appears to be similar to JSON, but there are a few differences. Specifications of what type of device is done with a hex value. This value would determine what states the device is broadcasting. Moreover, the information regarding which network that a device belongs to is encoded in the same manner. Without the documentation of this, the intercepted messages would not be as useful to a forensic investigator. If no useful data can be obtained through the intermediate devices, then the last option would be to obtain it from the cloud.

*Cloud services* as the final resting place for sensor data. Depending on the policy set forward by the provide, the data could be stored indefinitely or for a short duration. However, due to issues with jurisdictions to which the provider is located at. This could result in the forensics investigator being barred from obtaining the required evidence, because foreign laws might protect providers against having to comply with seizure warrants. However, if the cloud service offers a platform for monitoring it is possible to obtain some information, granted that law enforcement can obtain the proper credentials. Smart device APIs that connects to a cloud can be accessed remotely, with software or tools. Depending on how the API is secured by its provider, it could mean that access is only granted to those who can provide the appropriate authentication. For instance, the Things Network is a commonly used API that supports gateway devices, such as LoRA gateways from Mikrotik. Their method of allowing API access, requires that the user provides three pieces of information: the application key, application id and device id. Without these credentials, no access i given. However, with the proper access, the data can be access, as well as logs to previous data. Provided that access is given to the cloud service, the next phase would be to obtain information. Ref. [16] provides a comprehensive list of manufacturers and also lists which of the devices made by these manufacturers are obtained by their proposed data acquisition framework. Their proposed framework, however, does not exclusively gather from just cloud sources, but also from the devices directly or from the controllers. Moreover, the article further states that the main method of accessing cloud data is done so through a supplied Application Programming Interface (API). Among the results listed in the tables, only a few selected candidates were successfully accessed through the by its cloud interface. If the goal of the investigation is to obtain information off of a device that was generated in the past, the cloud would be best candidate to examine. Since, nether the sensor, nor the intermediate device keeps data outside of their volatile memory, the only area to which persistence storage would be the cloud. However, just obtaining the evidence is not sufficient in forensics. Integrity of the evidence is equally as crucial as acquisition, and IoT devices poses challenges here as well.

#### 4.3.1. System Preservation

Evidence integrity is crucial for forensic analyst to reach the appropriate conclusions about their investigation. Integrity of evidence entails that the contents of the evidence is not altered. Circumvention of integrity in this instance, refers to actions of intentional or unintentional obstruction, which leads to the evidence becoming ineligible for use by forensic investigators. There are three main categories of entities that pose a risk to the nature of evidence. The first group is the forensic investigators themselves. Methods and practices used to procure and analyze evidence is not suited for all instances of devices. When an established forensic method is used for IoT, there is no guarantee that the methods are forensically sound and the risk of data becoming useless. For instance, given a scenario where a suspect is implicated as a suspect due to their cellphone’s location data indicating their presence at the crime scene; however, whereas the CCTV footage suggests that the suspect was never in contact with the scene of the crime, but rather in proximity. This particular case would suggest the potential for additional evidence, discovered in the IoT domain, to be used as a means of altering the likelihood of a hypothesis. In this case, it is used to eliminate a proven hypothesis, due to inaccurate location data. A major problem that is related to evidence is the risk to forensic examiner’s and forensic analyst’s mental health, when exposed to harmful material.

#### 4.3.2. Evidence Searching

Regardless of what is considered important to a forensic analyst, in contrast to a police officer, the data found on a IoT device, still pose a significant value to the analyst. Regular digital forensic evidence, meaning what is considered evidence found on computers and mobile devices, can be strengthened or weakened by introducing evidence that originates from IoT devices. However, as IoT is not an established means to which evidence is procured by investigators, a couple of challenges emerges when IoT data is to be acquired for forensic analysis. One of these challenges is on the unfamiliarity and lack of methods to extract useful information. According to [19] the footing of forensic methodology in the IoT landscape is lacking. Stating that as more devices are connected to the Internet and being able to transmit data over it, the challenges of acquiring and handling the data for potential evidence will worsen. Researchers in [19] further listed the three primary categories in which data is stored in IoT. The first one being the smart sensors, the second one is the intermediate connecting devices including hubs, computers and routers and lastly cloud platforms that aggregates and handles sensor data. All three categories pose challenges to evidence acquisition due to different issues.

### 4.4. Event Reconstruction

Reconstruction of digital crime incidents from IoT devices can be a difficult task. In Germany ZITiS [20] has created a physical mobile phone library in which they acquired nearly all known models of mobile devices from allover the world to be used in digital forensics investigation. In case of IoT such endeavors are possible but due the the ever expanding IoT landscape achieving such thing would be very difficult and financially not feasible, as reconstructing a digital replica of a smart city with physical devices for forensic investigation is not piratical. Cyber ranges [21] can have a valid use case in this domain for creating the digital twin of the crime scene for crime event reconstruction.

## 5. Devices

### 5.1. A City of Devices

It has become more important than ever to integrate the fundamental processes in a urban environment with contemporary technological solutions. In pursuit of integrating information technology into a city landscape, a city aims to draw the benefits that the modern tech-solutions has to offer. One major advantage of introducing technology into a society would be for its sheer convenience. By introducing a tool as a means of completing a task, the overall difficulty or strain involved with such a task is reduced. Smart cities pose such as convenience in a multitude of activities, including automation of payment processes, which were introduced in china in 2019. In this instance, the payment is proceeded through a facial recognition system, that are facilitated by China’s recent roll out of a comprehensive camera surveillance system.

Smartphones are another means to which users can use smart technology to commute from point a to point b. Using a smartphones location tracking capability, mobile applications such as Uber, Lyft, Juno, ReachNow and Via can connect a passenger with a chauffeur. Furthermore, transportation services and payments can be done through Near-Field Communication (NFC) on a smartphone, thus making paying for a train ticket more convenient. In homes, appliances can perform automated tasks, which could make a morning routine for a person more feasible. For instance, a smart home can detect when a user wakes up and then the hub could initiate a routine, such as turning on the coffee machine, adjusting the thermostat or citing news and weather. In industry and agriculture, smart sensors also can aid in making different processes involved in these sectors more manageable. Water levels in the soil at a corn farm can for instance be monitored with sensors, and alert when the soil becomes dry. Industry could use sensors in warehouses to communicate with robots, such as the automation that was implemented in Amazon warehouses [22]. All of these are made possible through the use of sensors, which requires input from the user and based on that input would perform an appropriate task. There are several areas of a city in which smart devices can be applied. In order to determine what devices can be used in police work and forensic investigations, a division of the different areas of the city must be divided.

### 5.2. Small Differences, Sways Applications

Most cities were built with an established ruleset in mind [23], which facilitates how its areas are arranged, connected and divided, how buildings are structured and placed within each zone and so on. However, the buildings and facility that are built within each of the cities do differ to a reasonable extent. For instance, cities built in inn-lands on arid fields, would have a higher need to build farms to exploit its local resources. Cities built near rivers would likely build mills and bridges. Meanwhile coastal cities would have harbors and docks for trade and export. What is essential for a city is what makes a city, but some cities have specific needs that must be satisfied, therefore the application of IoT technology would differ as well. As point of reference in this article, no city is the same. Cities do have a plethora of elements in common, which is to be believed to emerge within every city, due to its necessity by its inhabitants. These elements are expected to be present within every city as a means to serve a demand by its residents or commuters. In particular, in urban areas, where the number of inhabitants reaches a significant number, it is not uncommon for these elements to be a given; a recreational, financial, educational or otherwise required facility that a reasonable portion of those living in a city is dependent on using to a varying degree of frequency. For instance, most cities do possess some means of providing medical services to its inhabitants, whereas the extent to which these services are covered, and capacity may vary. On the other hand, cities also strive to provide the appropriate level of education, where this also may vary between cities. This may be attributed to the factors, such as population, public/private funding, the location to which the city were built. These are some of the reasons as to why a city can provide its citizens with multiple variations of the same services, whereas some cannot.

Funding, again, can be limited due to the low number of residents, which is the reason as to why it is less likely to build a large hospital in the middle of a small rural town in Nebraska, as opposed to building one in the heart of a town in the state of Maryland. It is by that fact, that the roll-out and planning of a smart city must differ between each city, and those who wish to plan out such a feat must be able to see the minor differences in facilities and needs. Only then can a city be properly equipped with state-of-the-art Information Technology installations that would best fit the needs of the citizens who lives there. What should be taken from this section is that it may be tempting to treat all cities as the same, based on the core facilities that are found in almost every cities, per definition of such. However, even those similarities may be different, to the point where servicing those facilities with smart technology could be considered unnecessary.

### 5.3. Elements of a City

To provide a complete list of all the elements that is to be expected in a city would be too exhaustive for this project. What this section aims to achieve is to set the exception of the reader as to what is most likely encountered and what is occasionally seen within the confines of a city. As a starting point the most common element of a city i the inclusion of a transportation system. Methods and means of transportation would vary from place-to-place; however, it becomes quintessential for a city to have some sort of transportation in place to be able to get citizens from point A to point B. Data that could surface when introducing smart technology in transportation services, solely relies of the means of transportation.

### 5.4. Surveillance

A visual overview of the crime scene can provide an operator or a forensic investigator with crucial details, which could aid them in performing their tasks. A popular candidate in this category would be the CCTV camera. IoT technology does not leave behind the technology of the past. In light of the push towards developing smart devices for use in cities, has reinvigorated discussions about the possibility of allowing surveillance cameras and surveillance systems of having the capabilities of more thoroughly observing and analyzing the footage to which they are observing. Cameras for use in surveillance, were previously only anticipated to be used as a means of detecting presence of trespassers, or to be used as a means of proving guilt when applicable. For instance, traffic cameras [24] do to an eloquent degree, aid in a courtroom to prove that a perpetrator did or did not violate the traffic laws. The use of camera footage in conjunction with license plates and other information allows for this to be an effective tool of incrimination or vindication. Another example of this being the case can be seen in convenience stores and shops, where surveillance is used to catch possible shoplifters and robbers. The main purpose of surveillance cameras being that they can be used as a means of proving that something unlawful took place at the scene. It becomes a necessity to serve justice, especially when surveillance makes it challenging for the defendant to prove their innocence when they have been caught on camera.

However, modern communication technology, coupled with computer vision and machine learning, has allowed cameras to process more information as they are observing. Technologies that allows cameras to determine what entity lies within their field-of-view, has allowed for detection of human faces, objects and animals. In [25] it was shown that the use of machine learning techniques, a CCTV camera were capable of differentiating between different kinds of wildlife using Convolutional Neural Networks (CNN). Another example of entity awareness in CCTV systems were demonstrated in [26], where they demonstrated a way for cameras to search for faces, using cameras on a public transport system. In essence, the ability to use new technology to re-purpose the old, allows for new ways of using existing technology for purposes, to which they were not intended to be used for.

In China, the idea of using surveillance to enforce public order has been in the spotlight in recent years, due to the sovereign state’s introduction of a social credit system. This system entails that the Chinese citizens are given a score, based on their day-to-day behavior, where inappropriate behavior is penalized, while good behavior is rewarded. With this system in place, the idea is that civil order is enforced through the idea of impeding social pressure upon the citizens, with the goal of pressuring them to become upstanding citizens [27,28]. By social pressure, the norm is set that persons who surround themselves with others that have lower scores, will also be penalized with a lower score, for choosing to associate with citizens of a “*less desirable character*”. One of the main topics raised when discussing the social credit system and the surveillance system in China is on the extensive roll-out of smart CCTV systems. What sets these systems apart is that they are interconnected with capabilities of identifying a person with facial detection. This allows the Communist Party of China (CPC) is able to keep records of citizens whereabouts at all times [27]. Presence (proximity) sensors is used to detect the presence of movement within a confined area. There are a multitude of applications, to which appliances rely on the detection of human presence or movement [29,30], in order to complete its sequence of actions. For instance, the use of burglar alarm systems relies on the ability for a sensor to provide the alarm with information about the presence of individuals within a home, when the alarm is activated. Another application where sensor data is required is to automatically perform a task whenever human presence is detected. This can be seen, typically in cyber-physical systems, where escalators, sliding doors and entrance bells are used in conjunction with a proximity sensors to activate a sequence of responses based on detection.

### 5.5. Residential Devices

Home door locks that can detect whether a person does require to enter or exit a building has become more popular in homes. For reasons, such as security, given that some smart door systems have authentication capabilities, that only allow passage when an authenticated subject is present. Another aspect of security with smart door systems is the fact that it can allow for a more secure way of checking to see who is at the door. This would involve either the use of an intercom system or an interface connected to a camera that is placed on the other side, facing the entry to show who is at the door. In addition, convenience, since a smart door system also do possess a mechanism which automates the locking and unlocking of a door.

There are potential for investigating cases in smart homes, where information that is gathered by a smart door sensor can be used to investigate further into an anomalous incident [31]. In [32], it was proposed in their smart home test-bed configuration, that the data that is being obtained through a smart door system, can be used to investigate the events that led up to an incident. In their example, the state of the door lock during the fire incident is checked for whether the door was locked/unlocked during the fire incident, or whether it was opened during the event. In [33], the potential for use of smart door sensors in forensic investigations can be justified due to the timeline capabilities of door locks. In their research, they inferred the significance of time logging in door system events and what potential it could have for the forensic investigator in a timeline analysis. For instance, the use of timestamps would help the investigator in establishing whether the door was opened and when it was opened during a criminal event.

### 5.6. Wearable Devices

Given that most forms of communication take place on a handheld device, Smart phones are relevant subjects for investigation due to its relevancy in every person’s life. In same conjunction to smart phones, Smart watches holds similar data to what a smartphone does. They have also become a crucial platform for means of communication, either with apps that are running on a smartphone or through built in apps [34]. Health monitoring either from a smart watch or from a customized device, can monitor the health status of an individual who wears the device. Electronic cardiac regulators, such as the Pacemaker are another example of a wearable device in which useful information is stored. Pacemakers are used as a medical tool to aid patients with uneven heart rhythms; however, it has been shown that these can be circumvented. According to [35], certain models of pacemakers have been proven to be vulnerable to attacks, thus allowing for a cyber criminal to tamper with the device, which can lead to death of the patient. Another example of a self-regulating medical device is the smart insulin pump. It is used to measure the patient’s glucose levels and administer insulin to the automatically. Unfortunately, as with the pacemaker, this device is also shown to have weaknesses. Researchers in [36] disclosed that there existed three vulnerabilities on the Animas OneTouch pump system. These three vulnerabilities allowed for eavesdropping, replay attacks and circumvention of the pairing process between the pump and its remote control.

### 5.7. Environmental Sensors

**Temperature sensors** are used in a wide range of applications, stretching as far as the use in industrial solutions, to agriculture and domestic settings. One of the main attributes as to why temperature sensors are used across various environments and industries is that it measures an important environmental value. For instance, in use in agriculture, more specific in a hydroponic growing facility, the use of thermal sensors are used to measure the levels of heat in relation to the room. With these measurements, the farmers are able to keep control of the exposed heat to their plants and can readjust water levels when required. Another example to which temperature is used is when indoor thermostats are to regulate and maintain their configured temperature settings. Lastly, a temperature measurement can be used in a fire suppression/alert system to which the sensors are used to signify the probability of there being a fire present in the building. In similarity to a temperature sensor, a **humidity sensing** device can monitor the density of water particulates in the air and is often used by weather stations to detect outdoor air humidity. Another measuring sensor for air quality is the various forms of **air quality** sensors. Depending on what type of gasses the sensors is capable of detecting, an air quality sensor can be used in applications, such as detecting presence of CO2 in the air when there is a fire, and the presence of other hazardous gasses. The application of these sensors can be applied to detecting leaks, possibility of illegal drug laboratories, help in determine a fire present and so on.

### 5.8. City Infrastructure

On the transportation side of a smart city, there are a few strides in recent years, made possible with the use of smart devices. Location tracking and near field communication are two particular technologies that has impacted the **transportation sector** in the last few years. Given that being able to track the location of one’s transport has proven to have impacted the way one use transportation serviced today. Previously mentioned companies, such as Uber, Lyft, ReachNow, Via and Juno are among some of the startup companies who leverages location tracking and smartphones to connect passengers and drivers. In public transportation, trains and the metro are gradually integrating elements of smart solutions into their business structure. For instance, the state of California implemented a NFC ticketing system, to reduce paper waste generated by commuters. Smart traffic management systems using computer vision and artificial intelligence, have been proposed in [37] where cameras could be used to detect wheel-chair users and provide them with more time to get across a road crossing.

## 6. Universal Internet of Things Format

A smart city can contain a plethora of different kinds of device. What types of devices a city has depends on what the city considers to be important. As discussed previously, the types of devices that can be seen in a city can range from a strongly interconnected one, or a city where smart technology has little to no influence. Regardless of how a city is built or the extent of embrasure that one has of smart technology, the coverage of a city that smart technology has is comprehensive. All listed devices previously is by no means a complete list of all types of devices that do exist today. However, the purpose was neither to provide one, rather to illustrate some conceptual devices that do exist and how they can be leveraged in law enforcement. Furthermore, the extent to which these devices do provide law enforcement with evidence is neither a complete list. The nature of information and how it can be procured by law enforcement and used to form a hypothesis, solely depends on the creativity of the examiner and the prerequisite evidence that are present at the time. Information on a later stage can be used as evidence in different cases, where they apply. It is not a guarantee that the same data source can be used when investigating different cases. Given below is the proposed system for integrating heterogeneous data from such devices.

### 6.1. System Architecture

**Figure 1:**
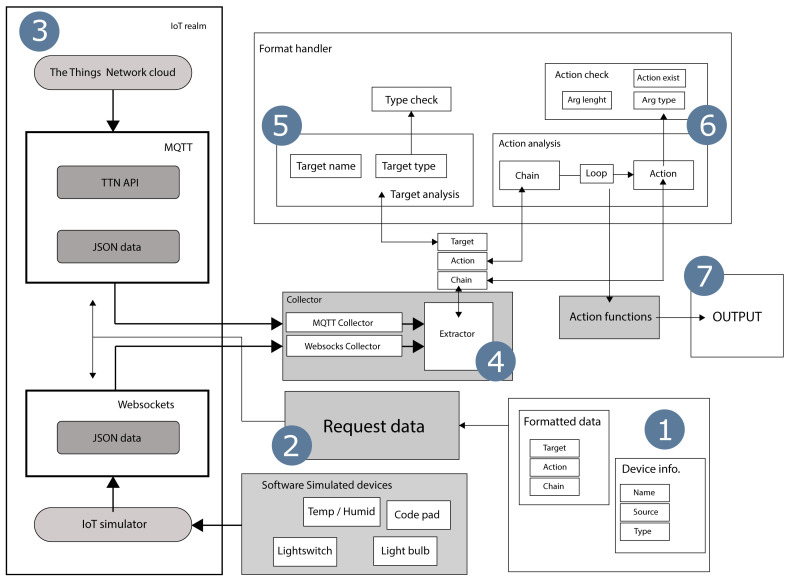
System architechture.

The Universal Internet of Things Format (UIOT:FMT) comprises of several components, where each part makes up the whole process of obtaining, formatting and post-processing the data. In the initial phase of this proposed architecture, the predefined formatting data is collected and loaded into the program. These formats will serve as recipes for how incoming data is interpreted and handled. The second, third and fourth component is the phases that involves the collection of data. Currently this component supports the use of Web-Sockets and Message Queuing Telemetry Transport (MQTT). The former is a test implementation done in conjunction with IoT device simulations, while the latter is an implementation done to collect data from The Things Network (TTN) API. Collected data is then being processed by the program, as shown in the fifth and sixth step, where the format specification for post-processing is performed. Here it is proposed to implement a system, where the JSON formats control what the program does to the data, after is has been obtained. Two introduced concepts shown in this paper is Actions and Chains, where Actions are singular function calls with data as input and Chains being a consortium of Actions sequenced in order. The overall system architecture is presented in Figure 1.

### 6.2. Data Format

The Universal Internet of Things Format (UIOT:FMT) is based on the well-known data serialization format JavaScript Object Notation (JSON). Given that JSON’s flexible nature and its low demand on computing resources when being handled by a computer program, makes it an ideal candidate for this proposed framework. Since data streams from smart devices would suggest the demand for storing data in different forms, the introduction of JSON would allow for all data to be properly assembled into this format. When compared against other contemporary serialization formats, such as Extensible Markup Language (XML), the use of JSON would allow for storing all data, even data in the form of arrays and matrices. When a user defines the formatting structure for the program to use, the conventions are the same as used in JSON, where the key and value is entered and separated by a colon (:). However, there are a few differences in syntactically typed.

First difference as observed in Listing 1 is that the value field holds the specifications needed to identify and locate the required data entries. There are three primary labels that must be known: *key*, *path* and *type*. Forward slashes (/) are used to delimit the labels, while colons (:) are used to separate the label from the argument and lastly if the argument comprises of several parts, a full-stop (.) will be used to separate these components. A path or a key label is used in this format to locate the desired entries from the smart devices. When a key label is invoked, the program will recursively look through the incoming data to see if the key exist. This method is slow, as it requires the program to look through all fields until a match is found. Moreover, if there are more than one entry with the same key, the first found instance will be selected. A path label will alleviate these complications, by specifically defining the direction to which the program should search to find the entry. Paths are therefore more ideally used for larger datasets, while keys are more suited for smaller sets. The type label used here is to ensure that the program is able to determine what data type is to be expected. If the incoming data’s type does correspond with the specified type, the program will attempt to use type assertion to convert the type accordingly.


**Listing 1.** Format example.

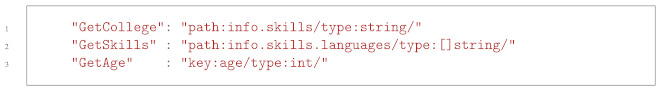




### 6.3. Post-Processing Functions

Some data input from devices might not just be different in the way it is structured by its origin, but its very nature might also dictate that the data is represented differently that other data entries. An example of this can be seen in temperature sensors, where the output of the sensors could either be in Celsius or in Fahrenheit. Another instance could be that the defined data-type of the data entry is different to what is expected, for instance an integer represented as a string, a Boolean value represented by 1 and 0. Alternatively if the output of this framework were to be used in a different program, the data representation required for that program might dictate that its input is formed in a particular manner. Therefore, in order to use the output, there must be a set of methods to ensure flexibility in how the data is treated by the program.

This research introduces two new concepts: first idea is to define *processing actions* to handle the data once it has been retrieved. An action comprises of a key and value, where the key signifies that it is a Action-call, while the value represents the function that is being called. A processing step that has already been discussed is the type conversion function, where the program will perform type-checking and conversion. However, when the output must be converted further, the functionalities of Actions is needed. In Listing 2, the actual output is re-converted to a float before it is forwarded to another node.


**Listing 2.** Action example.

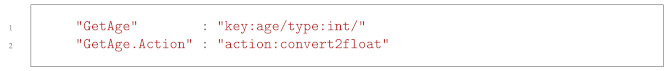




Another important aspect of Actions is that the user can specify arguments for Actions that requires additional input. In Listing 3 the syntax for specifying arguments, requires a parenthesis enclosure, a s-type assertion and the input argument. The s-type is proposed here to enable the program to distinguish between the types supplied by the format file and the types required by the action function. For instance, a reference to another collected variable would be denoted with *sref*, while the current variable is simply *self*. All literal values (“string”, ‘c’, 69, false and 33.3) will be denoted by their corresponding s-type notation (sstring, srune, sint, sbool and sfloat64).


**Listing 3.** Action with arguments.

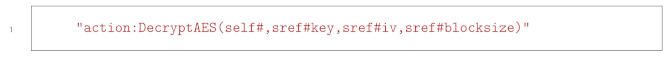




Chains are introduced in this framework to allow for use of more than one function per entry. The concept of chains works in the same fashion as Actions, although each separate link (Action) must be separated by a full-stop. An example as shown in Listing 4, a retrieved number might require that more than one arithmetic operation to be done to it, therefore a chain will be the best solution. One benefit of chains is that it is more flexible than hardcoded functions. Where operations are linked together in a chain, rather than pre-written into the program itself.


**Listing 4.** Chain example.

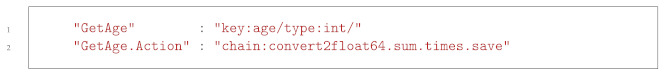




## 7. Evaluation

This research artifact comprises of two methods of communication, the first one is Message Query Telemetry Transport, which is the one used by the TTN Api hook, while the second protocol is based on websockets and is implemented through Golang’s standard library. The reason this evaluation focuses more on assessing its own performance against protocols, rather than assessing the formatting of these frameworks, is that measuring data value to a community would be subjective. Assessing the value of data as evidence for police, in comparison to the value of medical data for physicians or financial data for a stockbroker, is that there are uncertainties as to how such a value could be measured. Values of data has that inherent nature of being a metric that has no definite and established definition of what could be considered valuable to a profession. In [38] it was discussed that an evaluation that bases itself on metrics that are inherently subjective, are metrics that are prone to be established on biased terms. In Law Enforcement, such a bias would exist in conjunction with how data is perceived to be to the individual investigator and field operator. For instance, while forensics have well-known sources of data that could be used as evidence, phone-call history, chat messages, video surveillance footage, server logs and so on. The fact still remains that other types of data from lesser known sources and that are infrequently sought to be of use, could be applicable in some instances, while in other cases it does not. Intrinsically the value of data cannot be measured due to its subjective nature, while intrinsically this subjective metric would make the results of such a metric in one domain different from another. The candidates that are chosen to be used in this evaluation are based on research conducted in the area of creating a formatting framework for IoT data for various purposes. The target of these proposed frameworks is their use of Application Layer IoT Protocols and these will be used to compare against the protocols used in this program. Since this program uses two different types of protocols, which are applied in two different ways, the testing done on these cannot be equivalent for all metrics measured. The measured metrics in this case are in part based on performance measuring of the program and the reliability of the protocol in terms of being able to handle large amount of traffic. This way, the proposed program is assessed for scenarios, which reflects that of a real-world scenario.

### 7.1. Application Layer Protocols

There are a multitude of different protocols that can be used in transferring information between endpoints. Ref. [39] proposed a unified data framework for electronic health devices. Their proposed framework uses Contrained Application Protocol (CoAP) to tranfer data to and from the program. Another project that uses CoAP was proposed in [40] and later developed further in [41]. Their introduction to a formatting standard uses CoAP in conjunction with ThingSpeak API and Mobile Crowd Sensing. Other application layer protocols, such as Message Query Telemetry Transport (MQTT) is proposed in [6], where they implemented an intelligence gathering framework for use in a smart city. When these frameworks were evaluated in this section, the main target for their evaluation will be on the protocols that they use. However, in order to test the proposed framework against other protocols, a test scenario for this application must be created.

### 7.2. Testing Framework

To show that this program is capable of handling a multitude of requests at once, a test scenario were created to demonstrate the overall power of this application. Since this program uses a combination of Websockets and MQTT, the testing has to be done separately on these two protocols. The test devices used for the scenario are presented in Table 1.

The first test conducted on this program is a simulation of scenario where there a is high volume of activity at once. For this test, a concurrent network handling function were implemented observe how the program would handle a large volume of data arriving at once. Additionally, a second function were created that automatically generates a pool of randomly generated devices. These devices can be assigned one of the following four test devices: Temperature/Humidity Sensor, key-pad lock, lightswitch and Light bulb. The first device is a temperature and humidity sensor that are configured to broadcast random sensor readings to the program. The second device is a simulation of an output key-code entry that a user would enter into a keypad to unlock a door. Third device is a light switch that can either have the value of on or off to indicate the state of the light switch. The last device in this test stores the color values of a colored light bulb, thus simulating the same values broadcasted by a smart light bulb. What each of these devices are assigned is solely dependent on a random value that are assigned upon the initialization phase of each device. In total there will be approximately *2000 devices* created in this test scenario and for each of these devices, a respective function will spawn a concurrent routine to instantiate a websocket connection to the main program’s socket handle.

One important factor to take note of is that both the simulated client and server are run on the same host. In other words, these measurements do not take into account the extra time it would take for traffic to travel over a plethora of additional gateways. Moreover, due to the multitudinous amount of performance spent on creating new devices, randomly assigning new data to it and send it over to the server, there will be some additional overhead to the measuring of the program’s performance. Another important thing to take note of is that a single machine can only handle so much processing before a bottleneck occurs. Therefore an additional time penalty is implemented in the testing to reduce the overall chances of there being a bottle neck on the server side of this test bench. Each device connection is assigned this penalty value right after establishing a successful connection to the server.

The MQTT protocol that is being used in this program is used in conjunction with a API that limits the connection bandwidth between a client/device and the cloud. For that reason, the total number of devices that can exist on a cloud application is limited to a 100 devices. However, as the number of devices increases, the latency of response from the server increases as well, and therefore the reliability of issuing uplink messages from simulations becomes less feasible. For all intents and purposes, to test the performance of the MQTT is less conceivable so due to the limitations set by the associated cloud service. To address the performance of MQTT cannot be reliably done in the same manner and therefore related research must supplement the measurements used when assessing the MQTT aspects of this evaluation.

### 7.3. Performance Testing

To conduct a performance test on this program, the proposed testing program will count the overall time taken by the program to finish a single execution cycle. Since this program does not implement counting of all operations conducted across all functions in all files, the best option in this case is to observe the total execution time. As described above, the total requests made to the server from the client side is approximately 2000 devices, with some being expected to not send information, due to the limitations put on the current testing environment. In order to test this program properly, a single cycle (with 2000 devices) are repeated several times over on the same connection, to see if the duration skews towards lower or higher value in a different cycle.

The functions shown in Listing 5 illustrates how the start and end times of the program are captured by the test cycle. These are placed in the program to set the time stamp before the networking sequences are called and one after the sequences are called details of which is presented in Table 2.


**Listing 5.** Time difference calculation.

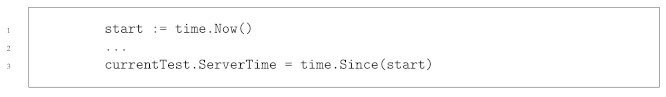




As shown, in the table, the duration recordings on ten execution cycles have similar values to each other. There are a few cases where the execution of the program takes anywhere from 0.5 to 1 s more than the values here, and this could be attributed to the volatile nature of the go routines that are not entirely running in parallel. Moreover, the added penalty time imposed on all the 2000 devices created, the additional 1 second would also be accounted for here. It cannot be counted off for the time being, as the nature of this delay is to make sure that the program does not crash, due to a limitation set on the total amount of concurrent connections allowed in go networking. Measurements of time efficiency of protocols, such as MQTT and CoAP is demonstrated in [42]. In this paper, the researchers attempt to measure the time of sending and receiving packages with these two protocols, among others. The testing involved sending packets of differing lengths and quantities, ranging from 25, 100, 250, 500 and 1000 bytes in length and at quantities of 5, 10, 20 and 50 consecutive packets. Using the measurements on the packets that are of the similar size to the packets generated by this project, such that the compared results are more appropriate. The generated packets in the program has the respective byte sizes.

The best option for a comparison would be to measure the packet timings for packets of 100 bytes in length. Could also be feasible to use the 25 bytes as well, but for simplicity’s sake, the 100 bytes packets will be the chosen packets to measure. However, from the results shown in this article, the time difference between 100 to 250 bytes is not that significant, nor is the time threshold between 25 to 100 bytes that significant different. However, this is only the case for MQTT and CoAP. In [42] the average time for these packets 0.24 s to 0.39 s for 100 byte MQTT packets at a quantity range from 5 packets to 50 packets. Meanwhile the time range for CoAP is between 0.23 s to 0.34. As shown there is a minor difference in speeds when juxtaposing these two protocols alongside each other. According to [43] the CoAP protocol is based on User Datagram Protocol (UDP), which does not care for ordering of arriving data and puts less stress on reliability of the messages to arrive. Its lack of Acknowledgements (ACK) of transferred data makes data arrive faster on the opposing end-point (Granted the packet reaches its destination). Now, the MQTT protocol does require that acknowledgment messages to be fed back to be able to ensure that packets do arrive as intended and this is slightly taxing on the overall efficiency with respect to duration. Moreover, these authors also demonstrated that regular HTTP protocol performs closely to MQTT on small packets, mainly due to the fact that MQTT does not employ additional threading and fault handling features. When measuring these protocols for performance times when sending a 100 byte packet 5, 10, 20 and 50 times over. Default byte sizes of non-randomized data entries are presented in Table 3.

Table 4 shows the results shown for the research results discovered in [42]. The results show that the time spent sending and receiving packets takes much longer to do for all of the other packets contrary to resulting average time gained in this work. The way average was calculated in this demonstration was done by summing the 10 results together and then divide by the number of cycles. Furthermore, the value of 1 were subtracted from the average to adjust for the 1 s penalty that was implemented to prevent goroutines from racing to finish too fast. Much of the reason this framework works more efficient is attributed to two plausible causes: the first reason for this is that the testing does not implement any form of assurance that information is sent, thus allowing for dropping packets. The other attribution to why these performance results comes out with such a low number could be that this program actively perpetuates the use of concurrency for all methods to reduce overhead on the main thread. While it is clear that the use of goroutines pose some advantage in the area of efficiency and lower latency, the question would then be if this is a safe way of transporting and handling data on a day-to-day operation. It might appear to be more efficient in some aspects, but this program does not take into account the packet re-transmission times, which is prevented by only allowing one transmission attempt. The next metric that should be addressed in this research is on the consequences of doing packet handling in such a manner.

### 7.4. Reliability Evaluation

As discussed previously in Section 6, with lacking sources of information about device fingerprints present for the program to use, there is little information that would enable this project to employ more automation. Therefore, this program cannot measure for this What the program can measure, however, is the extent to which it is capable of maintaining information that is fed through it. This test will therefore be conducted on the overall capability of the program to receive packets from the clients and how much of the information will not make it to the client. The first limitation imposed upon the program is a policy to which the packet is not re-submitted to the server if it failed to send. If the packet for any reason fails to send, the amount it presumably had sent is logged on the client side and stored. On the server side, all packets that has been received by the server is stored in a buffer with the number of bytes received.

After each of the components in the program has terminated their cycles, the total number of bytes logged on each side is calculated to find the percentage of loss.

(1) $$Packetloss\left(p\right)=\left(\frac{Device(P)-Server(p)}{Device(p)}\right)\times 100$$

This formula will measure the percentage of loss in bytes occurring in the program. The formula takes the fraction of the counted bytes on the server and the counter bytes from the devices. The way that these bytes are counted is done through the *net.write* and the *net.read* command, which both have a *bytes processed* return values. When network connections send and receives these bytes, the functions that handles these operations would confirm the amount of data sent and this is how the exact amount of data was found.

Listing 5 shows the resulting packet loss experienced over ten consecutive executions of the program. The number of devices is set to the default test value, with approximately 2000 devices broadcasting data to the server. No confirmation of delivery is supplied to the devices, nor is there a mechanism to enable a device to resend the data if a failure is detected. The only check conducted is the sending mechanism on the device side of the communication, which requires that the dispatch function supplies the amount of packets that were sent. The loss rate is calculated using the above-mentioned formula. It is shown that the current loss rate varies between the 10 cycles. In some cycles, the overall loss rate is below 1%, while in other cases the loss rate of the program can reach as high as 3.2%. This rate of packet loss in a testing that simulates a traffic burst over a short amount of time is deemed reasonable. In [45] it was concluded that if a system manages to maintain loss rates at a level of 3.6% or lower, then that system will be able to serve its purpose with minimal impact to its Quality of Service (QoS). The loss rate seen in this scenario could be attributed to the fact that the amount of traffic is large and because of the lack of re-transmission mechanisms, which causes the jump in loss rate.

According to [46] protocols such as CoAP that are based on UDP have a tendency to lose more packets when the amount of ongoing traffic is too high. They demonstrated this in a similar test scenario to the one demonstrated in this paper. They showed that the loss rate on different topologies that use CoAP will experience high rates of loss when the volume of traffic increases. This can further be seen in [47] where Packet Loss Rate (PLR) is directly affected by the amount of traffic on the destination. For the MQTT protocol, the same correlation can be drawn between reliability of packets and their timeliness. Ref [48] tested different 4-way handshake methods in MQTT to see where the reliability of information will change when activity on a connection increase. They showed, that when enforcing a regular 4-way handshake, the overall packet loss decreased slower than what it would when less strict handshakes were used. The percentage on a regular 4-way handshake on a wired connection to be around 0.20% when the load is around 16,000 bytes. When calculating the average packet loss rate of the program, using the same method that generated the cycle values in Table 5 amounted to a value of 2.18%. However, with the given discovery that large traffic generates lower successful transmission, the question would be what would happen if the delay penalty were to be randomized, instead of being a constant.

In Listing 6 it is proposed to modify the current testing framework, by changing the persistent penalty value that is being used on goroutines, to a value that is non-constant. The idea here is that when a random time delay is being introduced the distribution of which these routines are to proceed is more scattered. This would hypothetically in effect make the server experience less simultaneous traffic and become more capable of serving the devices as a whole. By introducing a randomized additional time penalty to each and every device the overall loss of packets is reduced and resulting in an average packet loss rate of 0.042% as presented in Table 6. Moreover, the overall number of devices could be increased to 5000 devices with this random delay. While 10,000 devices were also feasible, but with some cycles crashing on occasion. The changing of this value have shown that while the reliability of the program can be improved, but the overall speed is affected as a result.


**Listing 6.** Random time penalty.

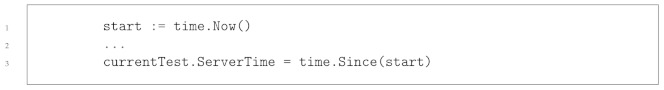




### 7.5. Functionality Comparison

Although this research focused on defining a structured data format for IoT device, such structured data has potential in digital forensic applications. One such potential was investigated by Garfinkel [49] in which the researcher developed *Digital forensics XML* DFXML language which enables the exchange of structured forensic data. DFXML contains the information related to forensic evidence such as document location, document type, file system information, and relevant information to forensic analyst. DFXML provides a necessary abstraction for understanding interchangeable forensics evidence. Compared to DFXML our proposed concrete syntax is JSON and our approach provides abstraction to format data from multiple IoT devices. We discussed the data format in Section 6.2 to highlight the differences between XML and JSON. In term of digital evidence DFXML provide more concrete prepossessed digital evidence information compared to UIOT which only focuses on raw data of IoT devices which can be used for identification of forensic evidence in future research.

## 8. Discussion and Conclusions

In order for the developed artifact be able to adapt to a diverse IoT landscape, there must exist a defined rule set which is used to conform the data when it is not of the correct type. It is proposed that an automatic type checking is enforced on the data when it has arrived, such that no misappropriated input is being processed and dispatched. Whether or not this is an appropriate measure is dependent on the person who are using it, as their intentions could require that data is outputted in a particular manner. For instance, a data scientist would require that data output is done in a particular integer/float type for use in machine learning, while on the other hand, some intentions for use of this program might not require any operations to be performed on the data at all. The proposed use of actions and chains would reduce the limitations set on what a user can do with the data, but it requires the user to define what they require of the data. Another topic equally as important is the ability to handle a vast amount of data at once.

Scaling the application to be able to handle as much information as possible is very important for this project, given that the area of application will contain a large number of devices that needs to be processed. In the evaluation it was identified that the problem with the proposed Websocket server is attributed to an imbalance between performance and accuracy. When the priority is set to favor performance, by making the program execute as fast as it can without interruptions, the reliability of that program would be severely reduced. An increase in reliability was achieved, but at the cost of performance, as the methods used to decrease lost packets involved setting a higher threshold on the total time that the devices had to wait. However, with the introduction of added delays at a random distribution, the overall load on the server was reduced. With a reduced load, the ability to scale up the number of devices was possible, which increased the number of total devices by a factor of five. An alternative proposal to handle the reliability problem would be to implement an acknowledgement of received package. For instance, instead of assigning random time delays between 3–10 s, the overall wait threshold could be reduced if the system has a way to verify transactions and re-transmit lost packets. However, this would only reduce some constraints on the performance, as the requirement fro re-transmission would require one or more additional dispatch attempt. With a proposed application that creates a format for use in a society in need for improved crime fighting, there are inevitable impacts that must be discussed.

In this paper, research wasconducted to discover whether it is conceivable to develop a solution that can create a common format for the heterogeneous data sets that are being created in the IoT landscape. It was shown that with the introduction of Actions and Chains, the user is more free to adapt the data to their specific needs. The capabilities of this program was tested and evaluated against other application layer protocols. It was demonstrated that this framework performs well against its competitors when there is a balance of performance versus reliability. As described in [1], this research work can be used for a proposed criminal activity detection system that draws some parallels with the fictional Artificial Intelligence surveillance system, from the TV-show *Person of Interest*. Both systems are based on an automated program, to which only the anomalous incidents get reported to the operators, leaving all other benign information a secret. Future research should provide the justification for deploying an intrusive automated surveillance application in a smart city and why it would be less damaging to one’s rights to privacy. When developing a detection system, it is also important to establish a baseline as to what is considered relevant for raising an alarm [2]. Given that this project is to establish an automated system to detect anomalous incidents that are detected through smart devices, it is also important to define what is considered to be anomalous. In undertaking this research problem, the researcher has the opportunity to discuss whether the next iteration of this detection system is to operate as a signature-based or an anomaly-based detection system.

## Figures and Tables

**Table 1:** Tested devices.

Device	Type	Protocol	Data
Arduino MKR 1300	Hardware	MQTT	Humid/Temp (float64 x2)
Lightswitch	Software	Websocket	On-state (boolean)
Light bulb	Software	Websocket	RGB (int, int, int)
Code pad	Software	Websocket	key 1–4 (int x4)
Temp sense	Software	Websocket	Humid/Temp (float64 x2)

**Table 2:** Time tests results.

Test	Bytes (Serv)	Time
1	130,986	2.1778662 s
2	135,021	2.1551524 s
3	137,697	2.1518888 s
4	129,978	2.1647948 s
5	132,836	2.1436297 s
6	128,906	2.1359591 s
7	133,325	2.6300981 s
8	138,069	2.1260972 s
9	129,365	2.5924328 s
10	132,910	2.5893287 s

**Table 3:** Default byte sizes of non-randomized data entries.

Data Entry	Bytes (Data)
Code Pad	75
Light Bulb	86
Light Switch	53
Temp/Humid	91

**Table 4:** Protocol time comparison.

Protocol	5	10	20	50
MQTT [42]	0.23	0.26	0.27	0.39
CoAP [42]	0.23 s	0.25 s	0.25 s	0.35 s
HTTP [42]	0.2 s	0.3 s	0.41 s	0.79 s
UIOT-FMT [44]	0.0011 s	0.0022 s	0.0034 s	0.0058 s

**Table 5:** Packet loss results.

Test	Bytes (Serv)	Bytes (Dev)	Loss (Byte)	Percent
1	137,496	139,647	2151	1.54031%
2	139,517	140,353	836	0.59564%
3	136,745	137,905	1160	0.84115%
4	130,142	132,780	2638	1.98674%
5	133,362	136,300	2938	2.15553%
6	133,127	133,519	467	0.34976%
7	127,592	129,836	2319	1.78609%
8	131,885	134,884	2999	2.22339%
9	133,955	135,656	1701	1.25390%
10	130,923	135,204	4333	3.20478%

**Table 6:** Packet loss result.

	MQTT [48]	CoAP [46]	HTTP [45]	UIOT	UIOT (With Randomized Time Penalty)
Loss (%)	0.20%	7.24%	3.6%	2.18%	0.042%
